# Case Report: Successful natural conception and delivery in a primary cancer survivor involving the reproductive, respiratory, and endocrine systems auth

**DOI:** 10.3389/fonc.2026.1763363

**Published:** 2026-03-06

**Authors:** Limei Tao, Shaojia Wang, Li Zhuan, Jian Xiong, Jingyu Yang, Jun Liu, Jiacong Yan, Yunxiu Li, Qin Xu

**Affiliations:** 1 Department of Reproductive Medicine, The First People’s Hospital of Yunnan Province, Kunming, Yunnan, China; 2 Department of Gynecology, The Third Affiliated Hospital of Kunming Medical University, Yunnan Cancer Hospital, Peking University Cancer Hospital Yunnan, Kunming, China; 3 Department of Reproductive Medicine, Yunnan Maternal and Child Health Hospital, Kunming, Yunnan, China; 4 Department of Thoracic Surgery, The First People’s Hospital of Yunnan Province, Kunming, Yunnan, China; 5 Department of Breast and Thyroid Surgery, The First People’s Hospital of Yunnan Province, Kunming, Yunnan, China; 6 Department of Obstetrics, The First People’s Hospital of Yunnan Province, Kunming, Yunnan, China

**Keywords:** case report, lung cancer, multi-disciplinary team (MDT), natural conception, ovarian borderline tumor, thyroid neoplasm

## Abstract

**Background:**

Natural conception in patients with multiple primary neoplasms (MPNs) is exceedingly rare, particularly those involving metachronous triple cancers of the reproductive, respiratory, and endocrine systems. This article reports the case of a young female patient who suffered from three primary neoplasms successively covering the three major systems of the ovary, lung, and thyroid, and achieved successful natural conception and delivery through comprehensive management by a multi-disciplinary team (MDT). This case provides a valuable reference for the diagnosis and treatment of similar patients.

**Case Description:**

A 28-year-old married female underwent right ovary-preserving radical surgery for bilateral borderline serous ovarian tumors (Stage IIIB) in February 2021. She received six cycles of postoperative leuprorelin therapy. She was diagnosed with microinvasive adenocarcinoma of the left lung (Stage IA1, *ERBB2* p.A775_G776insYVMA mutation) in May 2021 and underwent thoracoscopic wedge resection. The patient underwent radical surgery for papillary thyroid microcarcinoma (pT1aN0M0) in October 2022. She presented to the hospital with fertility concerns in March 2023. An MDT comprising specialists in gynecology, genetics, thoracic surgery, breast and thyroid surgery, obstetrics, and reproductive medicine held a consultation to evaluate the patient’s condition. The assessment concluded that all three neoplasms were in complete remission and that pregnancy did not increase the risk of tumor recurrence. Auxiliary examination revealed an anti-Müllerian hormone level of 1.40 ng/mL (only the right ovary remained intact). Hysteroscopy confirmed the diagnosis of chronic endometritis and endometrial polyps. The polyp was resected, and the patient received a 14-day course of anti-infective therapy with metronidazole and levofloxacin, after which she was guided by natural conception. She achieved a natural pregnancy in August 2023. The MDT provided dynamic monitoring throughout the pregnancy until April 2024, when she vaginally delivered a healthy female infant weighing 3090 grams at 39 weeks and 2 days of gestation. Postpartum follow-up revealed no signs of recurrence or significant abnormalities in the offspring.

**Conclusion:**

This is the first case of successful natural conception and delivery in a patient with metachronous MPNs involving the reproductive, respiratory, and endocrine systems. It establishes an MDT management pathway encompassing “determination of oncologic remission status, intervention for reversible fertility-compromising factors, and cross-trimester monitoring.” This confirms that natural pregnancy is safe and feasible for patients in cancer remission, with multi-disciplinary collaboration and rigorous monitoring. The absence of postpartum neoplasm recurrence and abnormalities in offspring provides a practical paradigm for fertility management in patients with MPNs.

## 1 Introduction

Multiple primary neoplasms (MPNs) are defined as the occurrence of two or more primary malignant neoplasms in a single individual, originating in one or multiple organs, either synchronously or metachronously, that are independent of each other, with no metastatic relationship (1). Based on the interval between neoplasm diagnoses, MPNs are typically classified as synchronous (diagnosis interval ≤6 months) or metachronous (diagnosis interval >6 months) (2).

With advancements in medical diagnostic techniques, extended survival of patients with cancer, and widespread adoption of early cancer screening, the clinical detection rate of MPNs has shown an increasing trend. Research data demonstrate that the proportion of MPN cases among all tumor cases varies significantly across countries: approximately 9.3% in the United States, 6.3% in Europe, and 0.60% and 2.67% in China (3). The pathogenesis of MPNs involves complex mechanisms, including common genetic susceptibility gene mutations (e.g., *BRCA1/2* and mismatch repair genes), exposure to shared environmental carcinogens (e.g., smoking and environmental pollution), and the potential carcinogenic effects of initial cancer neoplasm treatments (e.g., radiotherapy and chemotherapy) (1, 4).

MPNs originating in the female reproductive system are relatively rare. While dual primary neoplasms involving the ovary, endometrium, and cervix have been reported at some frequency (5, 6), triple primary neoplasms involving three distinct systems, the reproductive, respiratory, and endocrine systems, are exceedingly rare, especially in women of childbearing age. Women of childbearing age with MPNs face the unique and challenging clinical dilemma of balancing fertility with oncological safety. Standard neoplasm treatments, including radical surgery, particularly involving the resection of reproductive organs; radiotherapy such as pelvic irradiation; and chemotherapy agents with gonadotoxicity, can cause irreversible damage to the ovarian reserve, uterine receptivity, and the hypothalamic-pituitary-ovarian axis, leading to infertility (7, 8).

At present, there is scarce research on fertility management in women of childbearing age with multisystem MPNs. Such patients often undergo multiple surgeries and/or various adjuvant therapies. However, there is a lack of systematic assessment data and established guidelines regarding the cumulative gonadotoxicity of these treatments, overall physical capacity to tolerate pregnancy, and risk of different primary tumors being influenced by hormonal fluctuations during pregnancy, particularly tumors that are hormone-dependent or affected by it, such as certain types of lung cancer and thyroid tumors (9). Furthermore, a history of multiple abdominal surgeries may increase the risk of pelvic and abdominal adhesions, which could lead to potential adverse effects on fallopian tube function, ovarian blood supply, and uterine position, thereby interfering with natural conception (10). Additionally, the patients’ potential cancer-related anxiety and fear of cancer recurrence may significantly influence their desire for fertility and decision-making processes (11).

This report presents the case of a 28-year-old married woman of childbearing age who suffered from three primary malignant neoplasms within two years, involving the reproductive, respiratory, and endocrine systems. Such extensive multisystem implications are exceedingly rare among women of childbearing age, and published reports on the feasibility and safety of natural pregnancy following treatment are virtually nonexistent. This report provides valuable clinical data for this unique population. The management of this case encompassed multiple stages, including cancer treatment decision making in gynecology, thoracic surgery, breast and thyroid surgery, genetics, fertility assessment and preservation in reproductive medicine, pregnancy management in obstetrics, and assessment of offspring health. This highlights the importance of multiple disciplinary team (MDT) collaboration in a comprehensive management model from cancer diagnosis to childbirth and provides a reference framework and clinical insights for clinicians facing similar rare and complex scenarios.

## 2 Case description

### 2.1 Initial visit medical history (treatment course of three primary tumors)

A 28-year-old married female presented with an “ovarian mass” in February 2021. Gynecological ultrasound on February 9^th^ 2021 showed bilateral adnexal complex cystic-solid masses, the nature of which was undetermined and suspected to be ovarian cancer. Pelvic magnetic resonance imaging (MRI) on February 10^th^ 2021 indicated bilateral adnexal cystic-solid masses that were highly suggestive of ovarian malignant tumors, a slightly thickened peritoneum with nodules suggestive of peritoneal metastasis, and pelvic effusion. The following surgeries were performed on February 24^th^ 2021: left salpingo-oophorectomy, right ovarian cystectomy, reconstruction, appendectomy, omentectomy, and mesenteric nodule excision. Postoperative pathology confirmed bilateral ovarian serous borderline tumor stage IIIB, partially micropapillary subtype/non-invasive low-grade serous carcinoma, nodules in the greater omentum showing foci of non-invasive tumor implantation, and mesenteric nodules composed of fibroadipose tissue with mesothelial hyperplasia. Immunohistochemical staining on March 5^th^ 2021 showed CK (focal+), WT1 (focal+), CK5/6 (focal+), and Ki67 (-). The patient received six cycles of leuprorelin treatment from March to August 2021. She presented with a “pulmonary nodule” in May 2021. Chest computed tomography (CT) on May 11^th^ 2021 showed a ground-glass nodule in the apical-posterior segment of the left upper lobe (approximately 7 mm × 7 mm). The patient underwent thoracoscopic wedge resection of the nodules in the left upper and lower lobes and cautery division of the pleural adhesions on May 13^th^ 2021. Postoperative pathology confirmed left upper lobe microinvasive adenocarcinoma (diameter, 6 mm), perineural invasion (-), lymphovascular invasion (-), and visceral pleural involvement (-), and genetic testing revealed an *ERBB2* p.A775_G776insYVMA mutation with PD-L1 (-). She presented with a “thyroid malignant tumor” in October 2022. Thyroid ultrasound on August 30^th^ 2022 showed an abnormal echoic nodule in the left lobe (TI-RADS 4 B type). Needle biopsy on September 28^th^ 2022 was highly suggestive of papillary thyroid carcinoma. The patient underwent radical thyroidectomy for thyroid cancer on October 4, 2022. Postoperative pathology revealed a papillary thyroid microcarcinoma in the left lobe (diameter, approximately 5 mm). The patient recovered well after surgery. In February 2023, the patient presented to the Reproductive Medicine Department of our hospital due to failure to conceive after one year of unprotected intercourse.

### 2.2 Menstrual history

Menarche at 13 years old, cycle every 26–27 days, duration 7 days, moderate flow, and occasional dysmenorrhea.

### 2.3 Obstetric history

Both partners are in their first marriage, G0P0.

### 2.4 Personal and family history

Non-contributory.

### 2.5 Auxiliary examinations

An ultrasound scan on February 23^th^ 2023 showed intracavitary hyperechoic foci that were suspicious for multiple endometrial polyps, heterogeneous endometrial echo, and an anechoic cystic area in the right ovary (19 mm × 15 mm, 16 mm × 13 mm). Left ovaries were not detected. Antral follicle count (AFC) in the right ovary was 10 follicles and the result of anti-Müllerian hormone (AMH) was 1.40 ng/ml.

## 3 Diagnostic assessment

### 3.1 Diagnoses

Primary infertility, history of borderline ovarian tumor, lung cancer, and thyroid cancer.

### 3.2 Multi-disciplinary treatment

A multi-disciplinary consultation was conducted, comprising six departments, including Gynecology, Medical Genetics, Thoracic Surgery, Breast and Thyroid Surgery, Obstetrics, and Reproductive Medicine on March 8^th^ 2023. The team unanimously agreed that all three tumors were in complete remission with no signs of recurrence. Pregnancy did not increase the risk of recurrence. The patient’s ovarian reserve function was satisfactory; however, ultrasonography revealed abnormal endometrial echoes. Pre-pregnancy assessments, including hysteroscopy and evaluation of tubal patency, should be performed 3–7 days after menstruation without sexual intercourse to determine the possibility of natural conception. In March 2023, hysteroscopy revealed patency of the right fallopian tube, and endometrial biopsy indicated chronic endometritis (CE). The patient was treated with oral metronidazole (400 mg twice daily for 14 days) and levofloxacin (500 mg once daily for 14 days). Semen analysis of the male partner was normal.

### 3.3 Pregnancy monitoring and outcome

The patient was confirmed to have conceived naturally on August 3^th^ 2023, with serum progesterone at 139.0 nmol/L and β-HCG at 1482 U/L. A follow-up test on August 11^th^ 2023 showed progesterone levels of 125.0 nmol/L and β-HCG levels of 27,524 U/L. Ultrasonography performed on the same day indicated an early intrauterine pregnancy, revealing a normally shaped and positioned gestational sac measuring approximately 13 mm in diameter without a visible embryo. The nuchal translucency (NT) scan on September 25^th^ 2023 confirmed a singleton intrauterine viable fetus, corresponding to 11 weeks and 5 days of gestation. NT measurements were within normal range. The patient spontaneously delivered a live female infant at 39 weeks and 2 days of gestation on April 4, 2024. The birth weight was 3090 g and no significant physical defects or diseases have been reported to date.

### 3.4 Follow-up

Throughout the pregnancy, the patient underwent monthly thyroid ultrasonography and thyroid function testing. Tumor markers were checked regularly and pelvic adnexal conditions were reviewed during each prenatal ultrasound examination. Chest CT was not performed during pregnancy due to potential risks to the fetus. After delivery, the patient was followed up every 3–6 months with thyroid ultrasound, gynecological ultrasound, chest CT, and tumor marker tests. The most recent follow-up, in August 2024, showed no significant abnormalities. The patient was admitted to the hospital with abdominal pain on August 26^th^ 2024. CT suggested incomplete intestinal obstruction. The patient was treated with nasogastric tube placement and supportive fluid therapy. However, the patient’s symptoms showed little improvement. Subsequently, exploratory laparotomy was performed and the patient recovered well.

**Figure 1:**
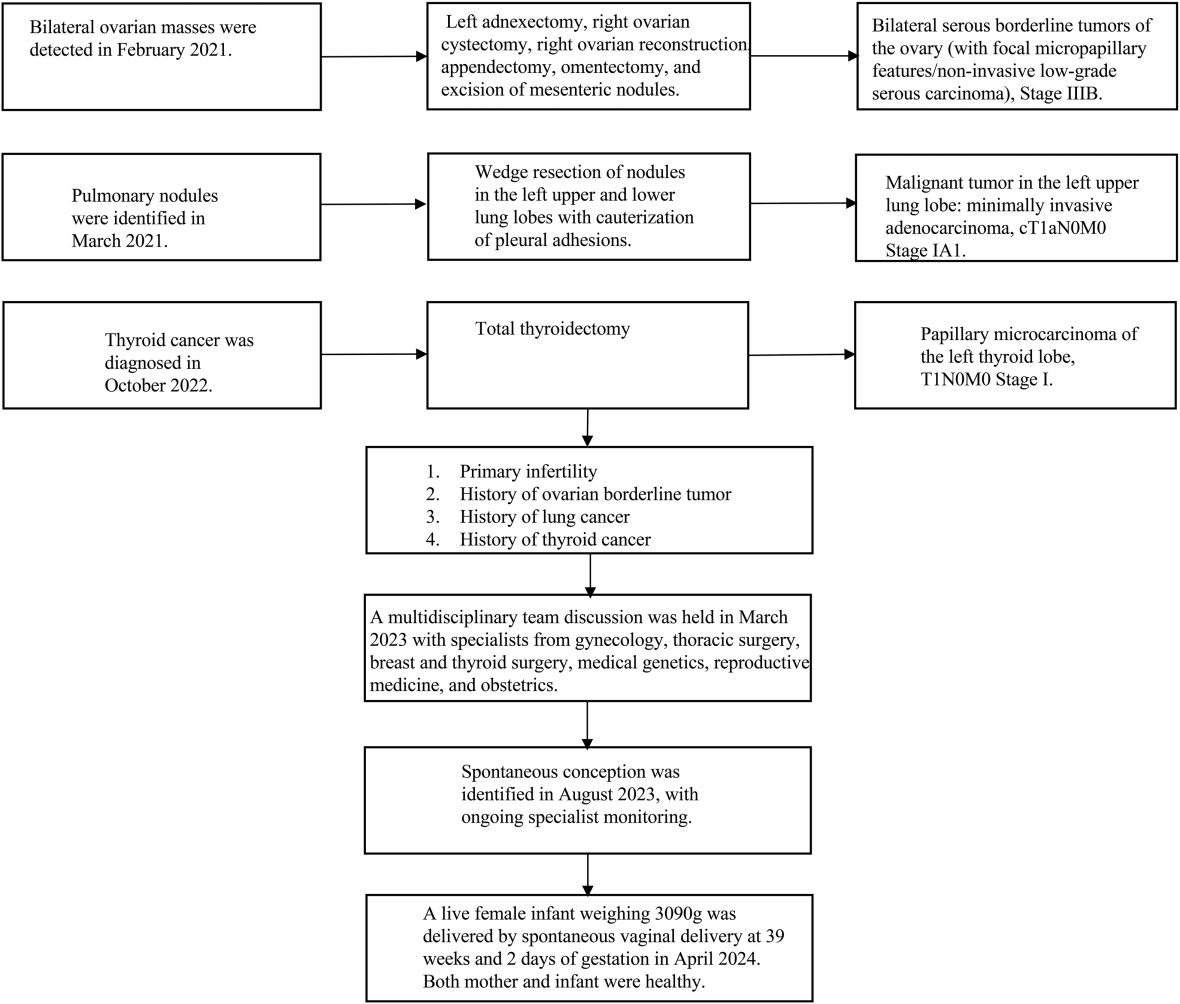
Patient treatment timeline.

**Figure 1** illustrates the timeline of patient treatment.

Written informed consent was obtained from the patient for publication of this case report, and approval was granted by the hospital ethics committee.

## 4 Discussion

Reports on fertility management of MPNs during reproductive age are extremely rare. Therefore, we recommend a multi-disciplinary and comprehensive management approach. This case involved three major cancers: borderline ovarian, lung, and thyroid. An MDT comprising specialists in reproductive medicine, gynecology, breast and thyroid surgery, thoracic surgery, obstetrics, and medical genetics was established for diagnosis and treatment. We conducted detailed discussions on key issues, including the prognosis of each cancer, its impact on fertility, the effects of pregnancy on tumors, fertility risks, and precautions for patients with MPNs. Our aim was to ensure the safety of pregnancy in this patient.

### 4.1 Borderline ovarian tumor and fertility

#### 4.1.1 Prognosis of BOT in women of childbearing age

BOT is a neoplasm of low malignant potential that predominantly occurs in women of childbearing age, with approximately one-third of patients diagnosed before the age of 40 years (12). Patients with BOT generally have a good prognosis. The 5-year survival rate for Stage I patients can reach 100% (13), and for Stage III patients, it decreases to 90-95% (14). In Stage I patients, the cancer recurs mostly as a borderline tumor, whereas Stage II-IV patients have a higher risk of recurrence as low-grade invasive ovarian carcinoma (15). Recurrence risk is associated with the surgical scope, pathological type, and high-risk factors. Unilateral salpingo-oophorectomy is associated with a lower recurrence rate than ovarian cystectomy and mucinous tumors have a lower risk of recurrence than serous tumors (13). Micropapillary patterns and invasive implants are significant risk factors for recurrence, necessitating long-term monitoring, with particular attention paid to tumor stage and pathological features (16). The patient in this case was diagnosed with Stage IIIB BOT of the micropapillary subtype, which is also classified as a non-invasive low-grade serous carcinoma. The gynecologist assessed that the patient was in a stable condition but had a high risk of recurrence, which could progress to carcinoma. Fertility attempts were permissible with rigorous monitoring and follow-up.

#### 4.1.2 Impact of BOT on fertility

Fertility-sparing surgery (FSS) is a key intervention in pregnancy. Unilateral salpingo-oophorectomy controls recurrence while preserving fertility and is superior to local cystectomy (13). Despite the relatively high risk of recurrence, given the favorable oncological prognosis of BOT according to the 2024 ESGO-ESHRE-ESGE guidelines, FSS can be considered for all patients of reproductive age with BOT, regardless of stage, provided that no invasive implants are detected (10). For patients with BOT of childbearing age who do not require chemotherapy, active attempts to conceive are recommended 3–6 months after FSS (17). A history of pelvic surgery may affect fallopian tube patency owing to pelvic adhesions and may have potential adverse effects on tubal function, ovarian blood supply, or even uterine position, which impact fertility (10). Reproductive specialists should provide optimal pre-conception advice based on the woman’s ovarian reserve and tubal patency as well as the male partner’s semen analysis, recommending either natural conception or assisted reproductive technology. Research has indicated that natural pregnancy rates can reach 60% in patients with BOT after FSS (18). In this case, the patient underwent a left salpingo-oophorectomy, but the right fallopian tube was patent, and the male partner’s semen analysis was normal; therefore, natural conception was feasible.

#### 4.1.3 Impact of pregnancy on BOTs

Studies suggest that hormonal changes during pregnancy do not significantly increase the risk of BOT recurrence, and that the overall recurrence rate during gestation is low (19). However, given the unique biological behavior of BOT, multi-disciplinary close monitoring during pregnancy is still necessary. Since BOT has low malignant potential, management of pregnant patients is primarily conservative (20). Literature reports indicate that patients with advanced BOT can still achieve natural pregnancy following conservative treatment, and close follow-up of these patients showed that pregnancy does not adversely affect overall survival (15, 21). For patients with Stage IIIB disease who have successfully conceived, constant vigilance against high-risk factors, such as the micropapillary subtype, remains imperative. Overall, the effect of pregnancy on BOT progression is limited, and personalized management and long-term follow-up are indispensable (22).

### 4.2 Lung cancer and fertility

#### 4.2.1 Prognosis of lung cancer in women of childbearing age

Lung cancer in women of childbearing age is predominantly non-small cell lung cancer (NSCLC), with adenocarcinoma being the most common type (23). A nationwide study in Denmark showed that female patients have a significantly better prognosis than male patients. Among the 14,635 individuals included, 50% were male (n=7,322), and the median overall survival for female patients with advanced disease was 8.8 months compared with 7.0 months for male patients (24). The 5-year survival rate for early stage NSCLC among Asian/Pacific Islander women is as high as 77.8%, which is significantly higher than that in other ethnic groups and may be attributed to higher surgical rates and favorable tumor biological characteristics (25). The patient in this case had minimally invasive adenocarcinoma of the left lung (stage IA1), classified as “localized early-stage” lung cancer. After anatomical lung resection, the patient has a relatively high survival rate and is currently in complete remission. Therefore, pregnancy attempts should be considered.

#### 4.2.2 Impact of lung cancer on fertility

Pregnancy co-occurring with lung cancer is relatively rare, and treatment decisions require a fine balance between effective control of the maternal tumor and maximization of fetal safety. Chemotherapy is considered a relatively safe option for patients in the second or third trimester of pregnancy who require systemic therapy (26). However, close monitoring of potential maternal and fetal complications, such as preterm birth, low birth weight, transient tachypnea of the newborn, and transient neonatal leukopenia, remains necessary (27). Compared to chemotherapy, the risks of radiotherapy to the fetus are more clearly defined, particularly when the radiation field directly involves the uterine area (28). Ionizing radiation from therapeutic radiotherapy carries potential risks of teratogenicity and carcinogenicity and is associated with fetal growth restriction, intellectual developmental delays, and increased risk of childhood malignancies (27). Therefore, radiotherapy should be performed with caution during pregnancy. Compared to patients receiving traditional chemoradiotherapy, those receiving targeted therapy for specific molecular mutations, such as in driver genes like *EGFR* and *ALK*, experience longer survival (27). For driver gene-positive NSCLC (e.g., *ALK*/*EGFR*), studies suggest that initiating targeted therapies alectinib, in the second trimester may reduce fetal risks (27, 28). Pharmacokinetic studies have demonstrated that the concentration of alectinib is significantly higher in maternal blood than in fetal blood, and no significant developmental abnormalities have been observed (29). Although targeted drug options exist for specific mutation types, such as *HER2* exon 20 insertion mutations, concerns regarding placental penetration and drug excretion during lactation must be addressed, and breastfeeding should be suspended during treatment (30). Genetic testing for targeted lung cancer therapy in this patient revealed *ERBB2* p.A775_G776insYVMA. This is one of the most common *HER2* exon 20 insertion mutations, with a clear tumor-driving mechanism and sensitivity to certain targeted drugs (31), which is of significant importance when formulating the treatment strategy. Furthermore, although reports are currently lacking for patients with lung cancer with a history of lung surgery, physiological changes in the cardiovascular and respiratory systems during pregnancy, such as increased tidal volume, elevated oxygen consumption, and elevated diaphragm, may increase the risk of dyspnea, decreased exercise tolerance, and other respiratory complications during gestation. Therefore, such patients require pre-pregnancy assessment and counseling, close monitoring of lung function during pregnancy, active non-pharmacological respiratory interventions (e.g., pulmonary rehabilitation), and consistent MDT management to ensure the safety of both mother and fetus.

#### 4.2.3 Impact of pregnancy on lung cancer

The effect of the hormonal environment during pregnancy on lung cancer progression remains unclear. Related studies indicate that estrogen receptor (ER) and progesterone receptor are differentially expressed in lung cancer (32). ERβ-positive tumors may influence progression through genomic (promoting proliferation via activation of the PI3K/AKT pathway) and non-genomic (rapid activation of MAPK signaling) pathways (33). Endogenous hyperestrogenism may reduce the risk of lung cancer by regulating aromatase activity, promoting the conversion of androgens to estrogens, and inhibiting the release of pro-inflammatory factors (34). However, multi-omics analyses have shown that among non-smoking patients with lung adenocarcinoma, exogenous estrogen exposure promotes tumor proliferation by activating non-genomic signaling pathways (e.g., PI3K/AKT) (35). During pregnancy, hormones may bidirectionally regulate lung cancer progression through receptor-mediated signaling pathways and immune microenvironment remodeling (36); however, the hormone-tumor interaction network requires further elucidation through single-cell multi-omics and cross-ethnic cohort studies.

Studies have shown that lung cancer diagnosis during pregnancy is often delayed and treatment options are limited (29). These patients are often misdiagnosed with pneumonia or asthma, and sometimes the respiratory symptoms reported by patients are attributed to increased respiratory resistance during pregnancy, leading to diagnostic delays and more advanced diagnoses (37, 38). To evaluate cancer progression during pregnancy, positron emission tomography or MRI has been reported to be safe for the fetus, with low radiation risk and the ability to provide accurate staging information (39, 40). The present case involved a patient who underwent surgical resection for minimally invasive lung adenocarcinoma (stage IA1), which was associated with a favorable prognosis. Due to concerns about radiation effects on the fetus, the patient did not receive any imaging examinations, (e.g., chest X-ray/CT), during pregnancy. Fortunately, postpartum chest CT showed no significant abnormalities. However, for patients with recurrent respiratory symptoms in early pregnancy, attention should be paid and imaging examinations should be performed promptly with biopsy, if necessary, to facilitate early diagnosis (26).

### 4.3 Thyroid cancer and pregnancy

#### 4.3.1 Prognosis of thyroid cancer

The global morbidity rate of thyroid cancer has steadily increased in recent years (41). According to the 2020 Global Cancer Burden Report released by the International Agency for Research on Cancer under the World Health Organization, thyroid cancer is the fifth most common cancer among women worldwide and second most frequent cancer in women aged <40 years (42). Thyroid cancer is often referred to as underlying cancer primarily because of its relatively favorable prognosis. The vast majority of thyroid cancers (approximately 80–85%) are differentiated types, such as papillary and follicular carcinomas, which typically progress slowly and respond well to treatment (43). Literature reports indicate that the 5-year relative survival rate for patients with thyroid cancer in China is 92.9%, which is slightly lower than the 98.6% rate reported in developed countries in Europe and America (44). However, the prognosis of thyroid cancer is influenced by multiple factors, with the pathological type and tumor stage being the most critical (45). Early diagnosis and standardized treatment are essential for maintaining a good prognosis. The patient in this case had a left-sided papillary thyroid microcarcinoma, which was low in malignancy and had a favorable prognosis.

#### 4.3.2 Impact of thyroid cancer on fertility

Thyroid functional status directly affects pregnancy outcomes. Hypothyroidism significantly increases the risk of miscarriage, preterm birth, and abnormal fetal neurodevelopment (46). Regular monitoring of thyroid-stimulating hormone (TSH) and dynamic adjustment of levothyroxine sodium dosage are necessary to maintain TSH within the target range (0.1-1.5 μIU/mL) (46, 47). Attention must be paid to the effects of specific treatments pregnancy should be delayed for 6–12 months after radioactive iodine (I-131) therapy to avoid embryonic toxicity from radiation (48). If newly diagnosed thyroid cancer during pregnancy shows no rapid progression or compressive symptoms, surgery can be postponed until delivery (49).

#### 4.3.3 Impact of pregnancy on thyroid cancer

Although elevated estrogen levels during pregnancy may promote tumor growth, studies have confirmed that estrogen has no significant negative effect on the progression of most thyroid cancers, particularly papillary carcinomas (49, 50). Guidelines from 2017 indicated (47) that survival rates in patients with stable thyroid function (TSH, 0.1-1.5 μIU/mL) were not affected by pregnancy, and there was no significant difference in the 5-year survival rate between those who become pregnant and those who did not (50). Low-risk papillary thyroid cancer, as in this case, progresses slowly, and the rise in estrogen levels during pregnancy has no significant adverse effect on its prognosis (49). Nevertheless, regular TSH testing and thyroid ultrasound are required to monitor the risk of recurrence. This case involved a low-risk patient, and breast and thyroid surgeons recommended routine follow-up with TSH and ultrasonography. Clinical decision-making should be stratified; if the tumor shows rapid growth during pregnancy or causes tracheal compression, surgical intervention should be considered during the second trimester (49).

### 4.4 MPNs and pregnancy

The pathogenesis of multiple primary cancers remains unclear, but genetic factors such as *BRCA* mutations may play a significant role. A study employing whole-genome sequencing revealed high genetic heterogeneity in patients with MPNs, providing a basis for individualized treatment (5). The safety of pregnancy and long-term follow-up outcomes in these patients are influenced by various factors including tumor type, stage, timing of treatment, and genetic background. Long-term follow-up of this case demonstrated that pregnancy did not significantly affect the prognosis of thyroid cancer, lung cancer, and BOTs, although continued follow-up over a longer period is still necessary. This case highlights the potential benefits of FSS, particularly for young female patients, enabling cancer therapy without sacrificing fertility, which is of significant social and psychological importance. Overall, the management of thyroid cancer and BOTs during pregnancy is relatively well established and has a favorable prognosis. However, lung cancer treatment during pregnancy remains challenging and requires further optimization of therapeutic strategies. Future studies should focus on elucidating the molecular mechanisms underlying MPNs, exploring precise treatment approaches during pregnancy, and accumulating long-term follow-up data to improve patient prognosis.

### 4.5 Application of MDT approach to MPNs

This case demonstrates the successful achievement of natural pregnancy in a patient of reproductive age with three primary malignant tumors through a phased decision-making and holistic management model implemented by an MDT. Initially, the MDT confirmed complete remission based on oncological safety assessments: 1) from a temporal perspective, the three metachronous primary cancers (February 2021: ovarian borderline tumor FIGO stage IIIB; May 2021: pulmonary microinvasive adenocarcinoma IA1; October 2022: papillary thyroid carcinoma stage I) met the criteria for MPNs (2); 2) pathologically, all three were low-risk subtypes (non-invasive omental implant in the ovarian tumor, no lymphovascular invasion in lung cancer, and no lymph node metastasis in thyroid cancer); and 3) molecular markers indicated low malignant potential (Ki67 negative, PD-L1 negative, and absence of *BRAF* mutation in the ovarian tumor, lung cancer, and thyroid cancer, respectively). Subsequently, fertility quantification identified both feasibility and challenges: preserved right ovarian function was observed (AMH, 1.40 ng/mL; AFC, 10 ng/mL), yet the ovarian reserve was reduced by approximately 50% compared to the median expected for her age [AMH, 3.0–4.0 ng/mL at 28 years (51))], along with the presence of an endometrial polyp. The MDT identified core infertility factors as the combined effect of anatomical and inflammatory factors, and a history of pelvic and abdominal surgery significantly increased the risk of CE (52) which impaired endometrial receptivity, and the polyp further reduced the likelihood of natural conception. Ultimately, a stepwise intervention strategy was precisely implemented: 1) first-line intervention was empirical antibiotic therapy (metronidazole and levofloxacin orally for 14 days); 2) second-line intervention was endocrine optimization by adjusting levothyroxine to maintain TSH ≤1.5 μIU/mL, mitigating the risk of miscarriage associated with hypothyroidism; and 3) third-line intervention was limitation of the natural conception window to six months, in line with ESHRE recommendations favoring natural trial for pregnancy when AMH >1.0 ng/ml (51). This approach led to natural conception within three months of active attempts, resulting in full-term delivery.

Innovation of the MDT-based strategy lies in three aspects. First, the team optimized the decision-making process. Although a BOT was initially diagnosed and at an advanced stage (IIIB), the team opted for FSS to preserve the right ovary instead of a standard hysterectomy with bilateral salpingo-oophorectomy. This approach addressed the most urgent oncological issue while preserving hope for future fertility. Second, preemptive risk management by correcting CE and optimizing thyroid function helped mitigate potential barriers to conception. Third, closed-loop management throughout the process established a continuum from cancer treatment to fertility intervention and then to antenatal monitoring, including tumor marker assessments every three months and regular obstetric check-ups to ensure both oncological stability and pregnancy safety. This case demonstrates that, for patients with complex multiple primary tumors, an MDT-led stepwise strategy from oncological safety to fertility assessment and then to targeted intervention coupled with holistic management is essential to achieve reproductive goals. In the future, fertility preservation strategies should be integrated into the initial phase of cancer treatment.

This case report has several limitations. First, the patient did not undergo genetic testing for cancer susceptibility, as recommended by the MDT. This is a fundamental limitation as it prevents determination of whether the three metachronous primary malignancies occurred sporadically or shared a common genetic driver, such as pathogenic germline mutations in *BRCA1/2*, *TP53*, *PTEN*, mismatch repair genes, or *RET*. Consequently, elucidation of the underlying pathogenic mechanisms is severely constrained. Moreover, the lack of genetic information meant that personalized genetic counseling could not be provided to the patient and their family. It also hinders the development of an intensified tumor screening protocol based on genetic risk and assessment of potential genetic risks for the offspring. Another limitation is the relatively short follow-up period, which contributes to uncertainty regarding long-term outcomes. The interval between the last cancer treatment in October 2022 and delivery in April 2024 was approximately 18 months, and the postpartum follow-up was even shorter. This timeframe is insufficient to evaluate the long-term risk of tumor recurrence or potential impact of genetic background on a child’s growth, metabolism, and future cancer risk. Continuous monitoring has become necessary over the years. Finally, the inherent nature of case reports limits the generalizability and depth of conclusions. The management approach adopted in this case, including the MDT process, key decision points, treatment, and pregnancy outcome, may not be directly applicable to other patients with multiple primary cancers considering variances in tumor characteristics, molecular profiles, treatment history, fertility status, comorbidities, and personal preferences. Although this experience may offer insights into the management of similar complex cases, it is insufficient to support the development of standardized clinical pathways or guidelines.

In conclusion, successful pregnancy in women of reproductive age with multiple primary tumors is rare, and the impact on fertility is dependent on cancer type. This case is unusual because of the occurrence of three metachronous primary cancers involving the gynecological, respiratory, and endocrine systems within two years in a 28-year-old woman who subsequently achieved spontaneous conception following FSS for advanced BOT. This experience offers valuable clinical insights into fertility preservation and the management of multiple primary malignancies. However, the generalizability of these findings is limited. Future studies should include more cases through multicenter, large-sample clinical studies to evaluate the efficacy and safety of different treatment strategies. Multi-disciplinary collaboration should be fully utilized to deliver individualized treatments. Long-term follow-up is also needed to monitor postpartum recovery, complications, tumor recurrence rates, and offspring health outcomes. Such efforts will help to inform future clinical decisions.

## Data Availability

The raw data supporting the conclusions of this article will be made available by the authors, without undue reservation.
